# Book Reviews

**Published:** 1832-02

**Authors:** 


### CRITICAL ANALYSES.

Quæ laudanda foreut, et quæ culpanda, vicissim

Illa, prius, cretâ ; mox hæc, carbone, *notamus*.—PERSIUS.

*A Critical and Experimental Essay on the Circulation of the Blood; especially as observed in the Minute and Capillary Vessels of the Batrachia and of Fishes.* By MARSHALL HALL, M.D., F.R.S.E., M.R.I., M.Z.S., &c.—8vo. pp. 187. R. B. Seeley and W. Burnside, London, 1831.

NOTWITHSTANDING the investigation of the powers that carry on the circulation of the blood has occupied the attention of physiologists, from the period of its discovery by HARVEY, many and contradictory opinions have been advanced, and are still maintained respecting them: some ascribing the sole power to the heart, some maintaining that the arteries perform an important part; others that they have no power in propelling the blood, but that their muscular coat and contractility, which are almost universally admitted, are merely to keep them in a state of tonicity, and that, if they contracted on the blood at all, it would rather impede than facilitate its progress; some also maintain that the capillaries are a principal agent, others that they have no influence; some that the veins are active agents, others that they are passive; some that inspiration is a powerful agent in moving the blood through the veins, others that it has no such power. The influence of the nervous system, too, on the circulation is a point on which there is much discrepancy of opinion: we need only allude to the opinions of LEGALLOIS, WILSON PHILIP, FLOURENS, and others, to shew that its real agency is by no means established. Considerable difficulty, too, exists in performing experiments to ascertain these points, inasmuch as there are many circumstances complicating them, and the accidental results have to be separated from those that are uniform, and follow as a consequence of the impressions made on the animal, and of the circumstances in which it is placed. This difficulty is so great, that Legallois remarked he obtained almost as many results as experiments.

Amidst all this variety and contradiction of opinions, any new facts tending to throw light on the subject, or the accumulation and examination of known facts, with the fair conclusions to be drawn from them, are particularly acceptable, in order, if possible, to set the question at rest. These are the points to which the well-known author of the work before us now draws our attention. His own observations have been principally confined to the lower orders of animals, which are peculiarly adapted to the purpose, as their parts are transparent, can be viewed for a long period together, and as they bear the necessary operations so well as to render the results much less complicated. As a preliminary step, he has taken great pains to describe a peculiar method of ascertaining the anatomy of the vessels which are the subjects of investigation. This occupies the first chapter of the work. Before proceeding with it, however, we would direct the attention of the reader to some excellent observations on the importance of the study of physiology, both general and comparative, and on the principles to be pursued in its prosecution. The great object is to avoid the infliction of pain, and therefore, as the author observes, "experiments should never be resorted to, when observation will afford the required information."

The study of the various cases of monstrosity and disease has, perhaps, not been sufficiently attended to: on this the author lays much stress, and shews that a proper attention to these will frequently prevent the necessity of performing experiments, nature having, as it were, already performed them. For the principles we must refer to the work itself; at the same time stating our belief, that, if they were observed, it would tend much to divest physiological research of the charge of cruelty.

In describing the anatomy of the minute and capillary vessels, Dr. Hall particularly adverts to the distinction between the different orders of vessels, viz. arteries, capillaries, and veins, which we must give in his own words.

"It is essential, in the next place, in describing the minute and capillary vessels and circulation, that we should attach distinct ideas to the various terms employed. It is, especially, quite necessary to distinguish the capillary vessels from the minute arteries from which they arise, and the minute veins to which they give origin. From the want of attention to the terms employed, much confusion pervades the descriptions of those authors who have treated of this subject.

"The minute vessels may be considered as arterial, as long as they continue to divide and subdivide into smaller and smaller branches. The minute veins are those vessels which gradually enlarge from the successive addition of smaller roots. The true capillary vessels are obviously distinct from each of these; they do not become smaller by subdivision, nor larger by conjunction; but they are characterised by continual and successive union and division, or anastomoses, whilst they retain a nearly uniform diameter.

"These distinctions are highly important: without them, the phenomena cannot be clearly described or understood. It is quite erroneous to speak of capillary arteries or capillary veins, or of the true capillary vessels as a venous network, as vessels containing a serous blood without globules, or as rather to be inferred than accurately seen. The last branches of the arterial system, and the first roots of the venous, may be denominated minute; but the term capillary must be reserved and appropriated to designate vessels of a distinct character and order, and of an intermediate station, carrying red globules, and perfectly visible by means of the microscope." (P. 17.)

Further on, when describing the anatomy of the vessels in the web of the frog's foot, he points out the mode of formation of the capillaries.

"The larger arteries first divide into branches; these subdivide into still smaller branches, which are also successively smaller than the trunk from which they proceed. At length the singular fact is observed of each of the two branches being as large, or even larger, than the vessel from which they originate. At this point there is an obvious and remarkable change in the appearance of the circulation; the course of the blood becomes of only half its former velocity, and the globules, consequently, instead of moving too rapidly to be seen, become distinctly visible. If the vessel be traced, it is next observed, not to subdivide, but to unite with other branches, and to pass into that distinct system and network of vessels to which I would restrict and appropriate the term capillary. The object of this peculiar distinction and character of the capillary vessels is very obvious: a more diffused and slower circulation is required for administering to the nutrient vessels or functions than that of the arteries: this peculiar character of the circulation is conferred at once, by the subdivision of the minute artery into branches of equal size with itself.

"Such is the phenomenon of the transition of the arterial into the capillary vessels and circulation. I have examined it repeatedly with every power of the microscope. It is however by no means difficult to observe; and it becomes still more obvious if the circulation be impeded or languid. It is, indeed, by rendering the circulation slow, by means which will be described hereafter, that the fact of the transition of the minute arteries into true capillaries is made most obvious. In the ordinary state of the circulation, the termination of the extreme minute artery is apt to be lost to view; and the change of rapidity in the circulation only adds to this obscurity by making the capillaries themselves more obvious.

"This is not the only mode of transition from an arterial to a capillary vessel. Sometimes one of the branches only, in the case of subdivision, becomes capillary, whilst the other remains arterial, pursues its course, and again subdivides variously; sometimes the artery becomes all at once singularly contorted, turning variously and giving origin to various capillary branches and the slower circulation.

"The capillary vessels themselves are situated intermediately between the minute arteries and veins. They are in a certain sense cylindrical, that is, of uniform diameter throughout their course; and however frequently they may unite and divide, and form anastomoses and even circles, they constantly retain the same size. The arteries and the veins, viewed merely in their trunk and extreme branch, or origin, on the other hand, may be considered as cones, the former becoming gradually smaller, the latter gradually larger, in their course.

"The minute arteries then gradually divide and subdivide; the veins unite successively into larger and larger roots and trunks, and frequently anastomose; the capillaries unite, divide, reunite, and redivide, and anastomose continually, so as to form a complete network of vessels of uniform character and dimensions." (P. 28.)

This mode of formation of the capillaries, their course and distribution, are, we believe, now first accurately described: at least, we have never met with any other such description. They have, it is true, been *supposed* to form an intermediate set of vessels, but their anatomy has never been described, or their commencement and mode of formation pointed out; consequently, nothing has been gained by the distinction.

The author then proceeds to describe the anatomy as it is observed in the different parts of the animals chosen for the subject, pointing out, as he goes on, the physiology arising from the distribution of the vessels, in relation to the structure of the part. In describing the mesentery, he adverts to a singular fact observed in the arteries and veins, viz. that they are covered with spots similar to those on the skin of the animal, this, Dr. Hall thinks, "may lead to some conclusion as to the continuous character of the external integuments and vascular texture." In the lung of the salamander, frog, and toad, Dr. H. points out an important difference in the formation and distribution of the capillaries, from that in the web, mesentery, &c.; the object of which is at once evident, as will be seen in the following comparison.

"If we institute a general comparison between the systemic and pulmonary circulation, we shall arrive at the following conclusions. The arteries in the former divide and subdivide at considerable intervals, until they become extremely minute; and, from the rapidity of the circulation, are only distinctly seen by the aid of the higher powers of the microscope; in the latter, the subdivisions of the minute arteries take place at the nearest points along its course, the arteries terminate abruptly; the branches assume at once the capillary characters. The veins are formed in a manner perfectly similar to that of the division of the arteries, in the systemic and pulmonary circulation respectively: the capillary vessels of the systemic circulation are far less numerous and more tortuous than those of the lung. It may be said, that, in the web, the vessels are adapted to support the nutrition and life of its various textures; in the lung, that the membrane is a mere scaffolding to spread the vessels which convey the blood in the fullest manner over its extensive surface." (P. 42.)

The only author who has described the vessels of the lungs is Malpighi; and in his work is a curious plate of the lungs of a frog, and also a figure of the vessels, as seen by him in the microscope: but it appears he had no clear idea of their origin or distribution; he merely represents the appearance they assume, and describes it as "quoddam rete mirabile."

In chapter the second, Dr. Hall proceeds to consider the powers which circulate the blood, and, before detailing his own observations, gives a sketch of the opinions of other physiologists who have investigated the subject experimentally. The great error in the reasoning of writers on the circulation seems to be in attributing too much, or the whole influence to one power, whereas, in reality, the circulation is sustained in a healthy and vigorous state by the combination of the whole; and it does not follow that, because it continues after one or more are abstracted, that these do not assist. If this were the case, we ought to conclude that the heart itself is useless, as the circulation is evidently carried on in fœtuses without any heart. The author first shews that the heart is the principal agent in the healthy circulation, its power extending, as he proves by experiment, through the whole series of vessels. He next shews that the arteries possess a considerable share in propelling the blood, arguing first from facts derived from cases of monstrosity and from comparative anatomy, and proceeding to shew, by experiment, that they possess a direct irritability and power of propelling the blood.

" A ligature was applied round the aorta of a frog. The circulation in the web, which was previously very vigorous, was almost immediately arrested, first in the capillaries, then in the veins. In the arteries there was a singular oscillatory movement of the blood for ten or fifteen minutes. The globules of blood proceeded slowly onward for some seconds; there was then, all at once, a rapid retrograde movement of the blood apparently through the same space. This oscillation was repeated: the globules of blood were again moved alternately in progressive and retrograde directions as before.

" It appeared to me that the artery gradually contracted, in successive portions, and slowly emptied itself by propelling the blood in a continued stream along its final branches; that it then dilated suddenly, and drew the globules of blood in a rapid retrograde course.

" During the first contraction of the artery, the blood would be propelled along the capillaries and veins. During the succeeding contractions and relaxations of the artery, the globules would merely oscillate, being driven forwards and drawn backwards alternately." (P. 78.)

The muscularity of the arteries, too, is shewn by the effect of water heated to 120° of Fahrenheit, which, as the author afterwards shews, induces contraction of the muscular fibre. The experiment is as follows, various parts being placed in the warm water.

" A mere fibre of a longitudinal muscle was shortened and made rigid; a portion of membrane or nerve underwent no change. An artery and vein were now placed in the warm water: they lay previously nearly equally flaccid upon a portion of glass: the influence of the elevated temperature was immediately seen in the artery, which became rigid and cylindrical; the vein suffered no apparent change. We have thus an important confirmation of the opinion that the arteries are possessed of a muscular, and consequently of a contractile tissue." (P. 80.)

On these facts and arguments the author observes,

"Still it must be confessed that none of these arguments are absolutely decisive: the facts afford no distinct proof of the muscular action of the arteries. The functions of these vessels are mingled in the cases of the acardiac fœtus and the acardiac animal, in the fish tribe and in the crustacea, with that of the capillaries; and, as we shall have occasion to repeat hereafter, unless we are enabled to separate these two orders of vessels, it is impossible that we should ascertain the function appropriate to each. The argument derived from the structure and the augmented action of the arteries, and the influence of an elevated temperature, is more distinct. The apparent effect of alternate contraction and relaxation of the artery after the ligature of the aorta, is certainly most powerful; indeed, it scarcely admits of a remaining doubt of the structure and function of the arteries. Still it is important that this possible doubt should be removed. This is finally effected, I think, by the fact which I am about to detail." (P. 81.)

This fact consists in the discovery of an artery in the frog and toad, which is distinctly seen to pulsate and move the blood it contains, after the heart and all the viscera are removed.* A plate is given of this artery, and we must refer for its description, &c. to the work itself. It appears to set the question at rest, and is the most direct proof of the independent action of the arteries, as every thing is removed but the portion of artery itself. We think, after the attentive perusal of this portion of the work, there can be no remaining doubt of the irritability of the arteries, and of their having a considerable share in carrying on the circulation of the blood: they seem to have a true peristaltic (not vermicular) action, and to act on each wave of the blood sent from the heart by successive and gentle undulations.

"The arteries are, indeed, a second heart in an elongated form: their function appears to be so perfectly performed in health, that all visible pulsation from their action is lost at their extreme branches. The blood is carried along at last by such gentle undulations, caught in their full force from the heart, but softened first by the elasticity, and then by the contraction of successive portions of the arteries, that its flow seems to become uniform. It is as the powers of the system languish, or when impediments to the action of the successive portions of the arteries exist, that the blood is seen to move in their minute branches in a pulsatory manner. (P. 85.)

The irritability of the true capillaries is next considered, and the author first points out the want of proof of their possessing any such power, and even doubts whether they be vessels at all, or mere canals. He observes, "The flow of blood through the capillaries appears, in every instance, to be effected and modified by

* We have seen Dr. Hall perform most of his experiments, and, among others, that here referred to. The pulsation of the arteries described continues powerful after the heart and all the viscera are removed.—Editor.

powers impressed upon it, of a character extraneous to any action of these vessels themselves. (P. 85.)

He shews that the principle of tension alone accounts for many of the phenomena observed in them, as the blood leaving them when the powers of life decline; its flowing into other channels, when its proper channel is obstructed, and its flowing in all directions to a point at which a vessel is opened. The same principle accounts for the apparent circulation in the minute vessels after the excision of the heart, or the division of the large vessels of the limb.

"This principle must never be forgotten in our experiments: otherwise, we shall imagine we see the effects of irritability in vessels, when, in fact, we only see the phenomena resulting from tension; or we shall imagine we see the loss of irritability, when it is but the effect of obstruction, and the power of tension yielding to those of the circulation. The effect of a ligature applied to a vein, and the effect of those substances applied to the web, which induce stagnation by altering the state of the interior membrane of the vessels, are examples of the latter kind. In both cases, the tension of the vessels or integuments yields to the forces which propel the blood, and the vessels appear enlarged.

"Incisions made through the membranes of the web or mesentery, always assume a circular or oval shape, by the operation of this tension and elasticity of these membranes; of which, therefore, there can be no doubt.

"This tension of the integuments is, I think, the source of many of the phenomena of the minute and capillary circulation." (*Pref.* p. xviii.)

The capillaries have hitherto been supposed to have a very considerable share in propelling the blood, and Dr. Philip, in particular, has argued for their irritability, and brought forward some experiments in support of his opinion; but Dr. Hall has pointed out many sources of fallacy in them, and concludes that the proofs hitherto adduced are by no means sufficient: he afterwards, in a postscript to the preface, goes further, and thinks that the phenomena observed on tying the heart prove that they have no such power. Its effect on the artery has been already detailed, and he asks, "if the capillaries and veins have the same power of irritability as the arteries, why do we not observe the same phenomena in them? If the capillaries possess a muscular power, why is the blood all at once motionless in them when the power of the heart is excluded? Must it not rather be inferred that, as the blood contained in these vessels is without motion, the vessels themselves are without automatic power?"

The explanation of the phenomena of inflammation is wholly dependent on a correct knowledge of the structure and functions of the minute blood-vessels, and the present explanation of *debility* of the capillaries, with increased action of the large arteries, cannot be true, if these views (and we see no reason to doubt their accuracy) be admitted: the question, therefore, is still open.

In considering the influence of expiration and inspiration, the author adopts, to a certain extent, the views of Dr. Barry, and records an interesting fact, that the effort of expiration in the frog, when the animal is struggling, instantly arrests the circulation; the actual expiration being prevented by a closure of the larynx. "It seems to be the reverse of the experiment of Dr. Barry."

In conclusion, the author considers the causes that modify the flow of blood in the minute and capillary vessels, which are particularly worthy the attention of those who wish to observe the phenomena of the circulation in the foot of the frog.

In chapter the third, the influence of the brain and spinal marrow on the circulation is considered. On this question the difference of opinion among experimentalists is equally great, as the author shews before detailing his own observations: much of this difference arises from the want of proper criteria by which to judge of the circulation and action of the heart. These are first pointed out, and the nature of the action of the heart, after its removal from the body, then considered. Legallois states that this action has no power to maintain the circulation, but that it is merely a movement of irritability. Dr. Hall, however, succeeded in seeing the circulation, though enfeebled, through the lung of the toad, after both it and the heart were removed from the body, and concludes that its action differs only in degree and power.

The result of the experiment of removing the brain and spinal marrow at once, which was performed on frogs and eels, by passing fine wire up and down the spinal canal, so as to destroy them successively, is thus stated.

"All these experiments appear to prove that the action of the heart is enfeebled from the moment it is deprived, at once, of the influence of the brain and spinal marrow. The connexion of this organ with the nervous system seems to be precisely of the same nature as that of the voluntary muscles: both possess a degree of irritability, independently of the large masses of the nervous system; both, if separated from these masses, gradually lose this irritability. The irritability is, doubtless, a faculty or property of the muscular fibre; yet it may become extinct without any obvious change in that fibre: its continuance or renewal depends ultimately upon the masses of the nervous system.

"The experiments which have been detailed seem to prove that, from the moment of the abstraction of the brain and spinal marrow, the irritability of the heart begins to fail. The circulation is first enfeebled, then lost, in the most distant parts of the system, then in parts less and less remote: the distance to which it extends may be aptly taken as expressive of the remaining power of the heart, the principal organ of the circulation." (P. 127.)

M. Flourens considers the circulation to depend on the medulla oblongata, and, as being the part which supplies the nerves to the respiratory apparatus, on that only; and states that every part of the spinal marrow but this may be destroyed, without influencing the circulation. To ascertain this point, Dr. Hall removed the medulla oblongata and brain, and the medulla oblongata only, in frogs and eels, and thus concludes:

"It is quite obvious, from these experiments, that the circulation no more depends upon the medulla oblongata than upon the medulla spinalis. I had previously determined that it does not depend upon the mere acts of respiration; for in the toad, in which respiration was entirely arrested, and from the lung of which plate VIII. was taken, the pulmonary circulation continued unimpaired during more than four hours." (P. 132.)

Le Gallois and Flourens also maintain that the circulation of each part depends on that part of the spinal marrow from which it derives its nerves: this, however, does not appear to be the fact, as the circulation in the tail of the eel is the first to fail after passing the wire down the spinal canal; whereas, the part of the spinal marrow supplying it with nerves cannot be destroyed, on account of the smallness of the canal. Dr. Hall relates experiments, too, where the lower portion of the spinal marrow being destroyed, the circulation in the web of the frog continued good. If the brain and spinal marrow be removed by small portions, at distant intervals, the circulation continues for a great length of time. Legallois observed the same fact, and explains it by supposing that the destruction of a small portion of the spinal marrow, insufficient to destroy the general circulation, diminishes it in the parts which derive their nerves from the portion destroyed, and that the circulation is thus concentrated, in the same manner as if a ligature had been applied round the parts. Dr. Hall offers a different explanation, as he finds the circulation is general, though enfeebled, and not confined to the parts whose nerves are derived from the remaining portion.

"In fact, if a portion of the nervous masses be removed, and if this be compatible with life, the animal is reduced to a lower degree in the scale of organised beings; it lives as a still lower animal; and it becomes capable, on this principle, of enduring new privations. I doubt not that, in this manner, the whole brain and spinal marrow may be removed, and that the animal may live, sustained by the mere ganglionic masses and the cutaneous respiration." (P. 137.)

We have not space to quote the experiments at length, but they deserve a very attentive perusal, as other deductions with respect to the irritability of the arteries, and the action of the nervous system on them, may be drawn from them. Whether the action of the heart and the circulation are wholly independent of the nervous system in the perfect animals, we think may still be reasonably doubted. The effect seems to depend very much on the suddenness of the removal, and the quantity removed. The effect of a shock, as produced by the difference between crushing and removing, which has been adduced by Dr. W. Philip, will not explain it.

In the experiments of applying stimuli to the brain and spinal marrow, from which certain conclusions have been drawn by Dr. W. Philip, the author's results are very different from that gentleman's, and, on comparing them, we should think them more correct; as it is difficult to imagine that the animal, being already rendered insensible, and an effect thus produced on its circulation by an agent which must act through the nervous system, would be excited by a reapplication of the same agent to the centre of that system. On the whole, we do not think that any very satisfactory conclusions can be drawn from this mode of experimenting. The effect of opium on the frog is curious, and worthy of attention.

In chapter the fourth, the influence of the other organs on the heart and circulation is considered. This is important in a medical and surgical view, and deserves the most attentive consideration. Every one is familiar with the effect produced by sudden injuries: this effect is clearly shewn by direct experiment, and in some instances when the brain and spinal marrow were removed; and the author concludes "that no physiological deduction in regard to the nature and office of any particular organ, can be drawn from an experiment of this kind. The only point really established is, that well-known, yet wondrous, connexion in the animal frame of every part or organ with every other part or organ."

"If the heart and circulation be viewed as independent of the brain and spinal marrow, yet impressible through them, this is equally true of their relation to the stomach or a limb: but, in fact, it is impossible to remove the brain and spinal marrow at once without immediately impressing the powers of the circulation, so that the motion of the blood in the capillary vessels immediately fails in the extreme parts of the system, and gradually in those placed nearer the heart. The powers of the circulation are, on the other hand, totally independent of any of the limbs; yet they may be suddenly destroyed through them." (P. 160.)

Inflammation of various organs, and its consequences, also produce similar effects.

In chapter the fifth, the effects of stimuli applied to the web are more fully considered. The author repeats his doubt as to the capability of determining the irritability of the capillaries by these means, and throws out some hints as to the explanation of the phenomena of inflammation; but, as he reserves this subject for a future opportunity, we only give one quotation.

"The immediate effect of the causes of inflammation is plainly physical, as well as vital, if it be vital at all. Inflammation itself is that process which is set up to remove the effects of irritation, and does not consist in the mere enlarged or debilitated condition of certain vessels, or the impeded motion of the blood contained in them." (P. 166.)

In chapter the sixth, Dr. Hall gives an account of a singular structure, illustrated by an excellent lithograph, observed during the course of his investigations, in the tail of the eel, which he has designated a "caudal heart:" this is sufficiently curious, and has never been before observed; it may lead to some interesting facts connected with the circulation, and he observes,

"Whether this structure be single or multiplied; whether it be peculiar to the eel, or common to other fish, as animals having a pulmonic heart only, or to other species characterised by the length of their bodies, are questions which I purpose to examine hereafter." (P. 173.)

In chapter the seventh, the author enters more at length into the effects of warm water on the muscular structure. This opens, too, a wide field of investigation, and we have no doubt will lead to interesting results: its effects on the Batrachia enable observers to perform many experiments on them without the infliction of pain, as it deprives them wholly of sensation and motion.

When we consider the difficulties attending microscopical investigations, and the patience and attention necessary in those pursuits, together with the trouble and time which must be devoted to the performance of experiment, we do not wonder that so little progress has hitherto been made in the department of minute anatomy. The secerning and exhalent vessels, the absorbent and incipient veins, are unknown and undescribed; the origins of the nerves, and the distribution of the minute and ultimate branches, and their relations to the muscular fibre, are interesting subjects of inquiry. The minute anatomy of the lower orders of animals, to which little attention has been paid in this country, remains to be investigated, and the organization, or want of organization of morbid structures, is to be determined. All these subjects must be pursued by the microscope; a wide field is open to inquirers; and it is probable that the next improvements in anatomy will be in consequence of its use.

We trust that Dr. Hall will not discontinue his investigations; for we know no man who carries on such inquiries with more impartiality, or who is better qualified to improve our pathological knowledge by experimental physiology.

*Essays on the Effects of Iodine in Scrofulous Diseases; including an Inquiry into the Mode of Preparing Ioduretted Baths.* Translated from the French of M. Lugol, Physician to the Hôpital St. Louis, by W. B. O'Shaughnessy, M.D. *With an Appendix by the Translator, containing a Summary of Cases treated with Iodine, either simple or combined with Opium, Mercury, or Lead; and Directions for Preparing the Iodurets of these Metals, and for Detecting the Adulterations of Iodine and the Hydriodate of Potash.*—8vo. pp. 218. S. Highley, London, 1831.

Lugol has made mankind his debtor, and that to no inconsiderable degree, for having reversed a cruel sentence, and cut off the entail from another of those ills that flesh is heir to; an ill which had so long and so obstinately defied the power of medicine, that its cure became despaired of, and its victims forbid to enter many of our public hospitals, as their desperate cases were not considered susceptible of professional relief; but, thanks to modern science, scrofula can no longer be esteemed an "*opprobrium medicum*," since Lugol has demonstrated, by a series of most satisfactory experiments, that many of its modifications are curable; i. e. submit as readily and as certainly to the treatment now proposed as any other chronic diseases are remediable by art.

We scarcely know a disease that hitherto we have considered, on the whole, more deplorable than scrofula, and its hereditary character formed one of its not least alarming features; for scrofulous persons are, in general, remarkable for beauty, and scrofulous females are, of all, the most prolific; hence it has so greatly spread, that we well recollect an old and experienced lecturer declaring that if it progressed the next century as it had done during the past forty years, hardly a family would be free from the taint, and men's heads, he perhaps somewhat hyperbolically added, would be seen almost falling off their shoulders in the streets.

When a disease is incurable, remedies on every side abound; i. e. when men of science fail to afford relief by rational means, men of art have an open field to impose on the credulity of the afflicted. Hence, for scrofula, specifics have not been wanting; specifics infallible in their failure only: of their potency and value a fair judgment may be formed, when it is remembered that the *manus regalis* was the most celebrated by far, and indeed the only one that for many succeeding centuries, from Edward the Confessor's time to the accession of the Georges, maintained its credit, all others being less depended on than the royal touch; and if the most valuable was so worthless, how *in*valuable must the rest have been.

It is curious, however, to notice, among the mass of absurdities referred to, that the very remedy now shewn to be so decidedly efficacious has, at various times, in various places, and in various unknown forms, been recommended for the cure of this disease, and that it exists in natural combinations nearly, in those proportions in which experience shews it can be most advantageously prescribed, and that even when iodine, the active ingredient of the bladder wrack and sponge, became separated, it at first was found less efficacious and manageable, merely because it was exhibited in less appropriate doses than it naturally existed in the Quercus marina or Fucus vesiculosus; and to a return to such moderate proportions does Lugol owe his brilliant success.

Iodine is one of the most important additions which the vegetable kingdom has made to our list of materia medica: we have watched its effects in several cutaneous diseases, as well as in bronchocele, and shall now watch its still more important prowess in the cure of scrofula. Were this a proper place for such an episode, we would fain have described a case of rupia, which for years withstood all other means, and which seems now to be yielding to the use of iodine; and, among other cases, we recollect one large goitre, which might also have been regarded as an *iodometer*, so regularly and rapidly did it decrease during its use, and again increase on its disuse, whenever the constitutional disturbance, probably arising from too large doses, forbade its further exhibition.

We have often regretted that, as many diseases once thought incurable, now are known to be curable, that "*incurable*" wards do not form a part of the general economy of our public hospitals, in which trials might be made for a sufficient length of time, and of a sufficient variety of means, to relieve, if not entirely to remove, those affections, the nature and the treatment of which are the least understood, and the least under the control of medicine in its present state, instead of at first forbidding the entrance of the most obstinate and least known cases, and then, should any protracted ones occur, discharging, by a kind of proclamation, or hospital delivery, all who have exceeded their six weeks or two months; unless the physician or surgeon will make a formal application for an extension of time; when he is often required to state *how much longer* it will take to effect a cure; a question which, in such cases as those we now advert to, it is often utterly impossible to give an answer. But "they manage these things better in France," and, had we hospitals for "incurables," we should soon have fewer incurables to need them.

It is time, however, to let the book before us speak for itself, and our first extract shall be from the report read by M. Dumeril to the Academie Royale des Sciences.

"MM. Serres, Magendie, and your reporter, proceed to lay before the Academy an account of a Memoir, presented by M. Lugol, doctor in medicine, on the Use of Iodine in Scrofulous Diseases.

"In the first place, we would remind our hearers that the scrofulous affections, long known under the names of 'cold humours,' or 'the evil,' constitute a class of those slow, unsightly, and often hereditary diseases, which strike despair into whole families, from the absolute rarity of their cure, and from the irremediable light in which they are regarded by the majority of physicians, and by the hospital regulations. Hence, also, the afflicted patients submit themselves to the illusive practices suggested by superstition; for, though medicine has successively tried all the remedies with which she is acquainted (the number, and even the absurd variety of which attest too strongly the want of a certain method of cure,) it must still be confessed that, up to the present time, an efficacious mode of treatment remained to be made known.

"Sometimes this disease is external and visible, and shews itself under the skin by swellings, which are slowly developed, become softened, burst, and remain ulcerated for a lengthened period, and thus produce callous and incurable scars; it takes its place in the substance of the integuments, which it renders deformed and disgusting; attacks the ears, the eyelids, the nostrils, and the lips, which become horribly tumid, or are corroded to such an extent as utterly to disfigure human nature.

"Sometimes, more deeply hidden, the scrofulous habit attacks the bones and their articulations, obstructs the canals which transport the lymph and chyle, or produces in the lungs, and most important organs, tubercles, which ultimately soften and degenerate into purulent centres, thus giving rise to serious morbid alterations in the living economy, which eventually yields to the effects of the disease.

"Such is an abridged view of the frightful malady to which M. Lugol, with zeal, perseverance, and success, has opposed a remedy, not absolutely new, but which had never previously been administered with so much method and precaution, to such a number of individuals at once, or with such evident and decided success.

"M. Lugol is one of the distinguished physicians attached to the Hôpital Saint Louis, the only hospital in Paris where a great number of scrofulous patients are admitted for internal treatment. This circumstance explains how, in the short period of seventeen months, from the 10th of August, 1827, to the 31st December, 1828, M. Lugol has been enabled to collect the detailed cases of upwards of 100 patients; in whom he, of course, found great variety in the seat and intensity of the disorder.

"Before your commissioners proceed to give an analysis of the memoir, they deem it right to declare, that they have not at all confined themselves to the scrutiny of its contents; but that they have seen, examined, and questioned the patients under treatment, and have also visited some of those reported cured or convalescent; that all the author's assertions have been found scrupuously exact; that many of the patients who were under treatment when the memoir was finished, have since been completely cured.

"Without restricting ourselves to the order followed by M. Lugol in his treatise, we proceed to make known its principal results.

"In the first place we may observe, that he uses two preparations of iodine: the one, exclusively intended for internal administration, is a solution of this simple substance in distilled water. The others are proper for external application, whether as ointments for ulcers, pomade for frictions, or watery solutions of varied strength, for collyria, lotions, and injections.

"The motives which have induced M. Lugol to employ by preference the aqueous solution of iodine, appear exceedingly plausible. So active a medicine can scarcely be administered in an hospital without inconvenience and uncertainty, except in the form of a drink. The alcoholic tincture and sirop of iodine present many disadvantages in the exact measurement and distribution of their doses, while a pint, or half a pint, of distilled water, containing in solution a little common salt, and a fixed quantity of iodine, affords us an easy, precise, and economical method of dispensing the remedy. Two degrees of this solution intended for the patients, and designated by the name of 'mineral water,' No. 1 and No. 2, (the first containing two-thirds of a grain, and the second one grain of iodine in solution,) have furnished the means of dosing exactly from day to day, and of recognising the effects of what was previously employed: thus, half of No. 2 is the first allowance, the entire of No. 1 the second, and, finally, the whole of No. 2.

"As to the preparations intended for the external treatment: these are unctuous substances of a certain weight, and associated in determined and successively increasing proportions with iodine, ioduret of potassium (hydriodate of potash), or with the proto-ioduret of mercury.

"These simple means have sufficed M. Lugol for the treatment and cure of numerous cases, twelve of which, selected from the different species of scrofulous affections, are described in the memoir. Three relate to ulcerated tubercles, cured in three, seven, and twelve months. Two cases are also described of ophthalmia and coryza, one of which yielded to a treatment of forty-six days, while the other was prolonged to the ninth month. A case of fistulous abscess deeply situated in the cellular tissue, has required nearly a year's care. Four cases are also recorded by M. Lugol of that frightful form of the disease most usually denominated 'dartre rougeante,' but which the author names the *esthiomenic* (or corrosive) scrofula. Finally, a case of scrofulous caries is detailed: this last form has generally been found very intractable. M. Lugol is only able to advance this single case of cure. It will be remarked, also, that the proto-ioduret of mercury was used, and that there still remains a small fistula as yet unhealed, but which appears to have a tendency to cicatrization." (P. 3.)

The cases referred to in the report we find are too numerous and too long for extract, although we had marked many for transference to our pages; but they are so interesting and convincing, that we recommend them to our readers' perusal; suffice it for us to make known the general results, and we fully agree with the author that "the importance of the facts related is great," for they contain proofs that iodine, properly administered, is not followed by any untoward effects of emaciation, &c. as when improperly employed; and that numerous cases of severe scrofula have been entirely cured, and others greatly relieved; to part of which he refers in the following summary.

"Eugene Chateau, Francois Poirè, Claude Michelot, Marguerite Bringer, are among the first scrofulous cases I treated. I did not place them under the same head on account of the cruelty of informing them that no hope was entertained of their cure.

"Richard, affected with a disease which had mowed down eight of her brothers and sisters, and who saw before her, in sad perspective, a scrofulous sister two years older than herself, and labouring under thoracic disease, with tubercles in her lungs.

"Adele Gandil, the offspring of a tubercular mother, and herself affected since the age of ten with scrofula in its worst form.

"Amand Olivier, under similar hereditary circumstances to Gandil, labouring under four fistulæ in the most dangerous vicinities, and which entitled us to apprehend the worst from the state of etiolation, debility, and emaciation, into which profuse suppuration of four years had thrown the unhappy patient.

"These facts, and a still greater number yet remaining to be described, place iodine in the rank of the most active and efficacious remedies which the art of healing possesses, its introduction into medicine should therefore be considered as one of the most precious improvements in that science.

"We have before our eyes patients attacked with scrofulous lesions, usually productive of a state of marasmus and colliquation, and which, under the influence of iodine, energetically resist the inroads of the disease. In some, even of this description, a marked tendency to cure is observable, too weak or transitory, it is true, to permit the encouragement of rational hope. Under this head are particularly placed cases of caries and of certain tubercular tumors of great magnitude, and which could be expected to yield but tediously to the influence of curative means." (P. 49.)

In the earlier cases, M. Lugol used an aqueous solution of iodine for internal administration; this he has subsequently changed for a combination of iodine with hydriodate of potash; it may, however, be the most advantageous plan to follow the example of the translator, and afford our readers a view of both the earlier and the later formulæ.

"*Ioduretted Ointment.*

| | No. 1. Old. | No. 1. New. | No. 2. | No. 3. |
|---|---|---|---|---|
| " ℞. Fresh lard . . . | lb. ij. | ℥ij. | lb. ij. | lb. ij. |
| Hydriodate of potash, | ℥iv. | ℈iv. | ℥v. | ℥v. |
| Iodine . . . . . | ʒiv. | gr. xij. | ℈xiv. | ℈xvi. |

"Afterwards I made use of a solution of iodine, which occasionally forms a valuable substitute for the preceding ointment, especially in scrofulous ophthalmiæ, and for the injection of fistulous canals.

"*Ioduretted Solution for external use.*

| | | No. 1. | No. 2. | No. 3. |
|---|---|---|---|---|
| "℞. | Iodine . . . . | gr. ij. | gr. iij. | gr. iv. |
| | Distilled water . | lb. i. | lb. ij. | lb. iij." |

The above solution the author has now abandoned for the following, which he finds "the most certain and least inconvenient method of prescribing iodine for internal use."

"*Ioduretted Mineral Water.*

| | | No. 1. | No. 2. | No. 3. |
|---|---|---|---|---|
| "℞. | Iodine . . . . . | gr. $\frac{3}{4}$ | gr. i. | gr. i$\frac{1}{4}$. |
| | Hydriodate of potash, | gr. i$\frac{1}{2}$ | gr. ij. | gr. ij$\frac{1}{2}$. |
| | Distilled water . . | ℥viij. | ℥viij. | ℥viij." |

"I commence the internal treatment with a half a grain of iodine; for this proportion I prescribe two thirds of the mineral water No. 1. In the second fortnight I gave the entire of this number; that is, three fourths of a grain daily, varying the dose within narrow limits according to the peculiarities of the case. During the fourth fortnight, or in the beginning of the fifth, I give a grain daily, and usually I continue this quantity to the end of the treatment. In some cases I have prescribed one grain and a quarter; still more rarely I have increased the dose to a grain and a half, but I have never gone beyond this quantity daily.

"Another and advantageous form of preparing this mineral water on a larger scale is, by first making a concentrated solution of iodine in hydriodate of potash, and then diluting it with a sufficient proportion of water; thus,

| | | |
|---|---|---|
| "℞. | Iodine . . . . . . . | ℈i. |
| | Hydriodate of potash . | ℈ij. |
| | Distilled water . . . . | ℥vij. |

"This solution contains one twenty-fourth of iodine; poured into sixteen pounds of distilled water, it forms thirty-two bottles of eight ounces of the mineral water No. 1. It is easy to understand that by diminishing the distilled water one fourth we compose No. 2, and by using three fifths of the quantity of water we obtain No. 3.

"Again, the concentrated solution now used serves for the administration of the remedy in drops once or twice daily, a mode of prescribing I frequently follow in my private practice. I commence by six drops given in the morning fasting, and six in the afternoon an hour before dinner, in half a glass of water flavored with sugar. Every week the daily dose is increased by two drops until it shall have reached thirty, or even thirty-six drops daily.

"For children under seven years old, I would recommend two drops twice daily for the commencement, to be increased gradually to five drops twice a day, morning and evening.

"From seven to fourteen years of age, I seldom order more than sixteen drops daily; I should not deem it prudent to exceed that quantity." (P. 166.)

"*Ointment of the Proto-ioduret of Mercury.*

"The following formulæ express the quantities of the ingredients in the several strengths of the ointment which I am in the habit of prescribing.

| | | | | |
|---|---|---|---|---|
| ℞. | Proto-ioduret of mercury, | ℈ij. | ℈iij. | ℈iv. |
| | Fresh lard . . . . . | ℥ij. | ℥ij. | ℥ij." |

"*Solution of Iodine for external use.*

"In my first memoir I only mentioned one solution intended for external use, but similar reasons to those which induced me to change the formula for internal doses have led to the use of the subjoined solutions, which are at the same time more permanent in their constitution, and uniform in their effects.

| | | No. 1. | No. 2. | No. 3. |
|---|---|---|---|---|
| ℞. | Iodine . . . . . . | gr. ij. | gr. iij. | gr. iv. |
| | Hydriodate of potash, | gr. iv. | gr. vi. | gr. viij. |
| | Distilled water . . | lb. i. | lb. i. | lb. i. |

"Injections may be made on the conjunctiva and the lacrymal passages with the solution; also, in cases of coryza or ozœna, it should be used several times a day, and is best applied by means of a syringe. In injections of the nasal fossæ, care should be taken not to direct too much of the solution towards the internal fauces." (P. 171.)

"*Rubefacient Solution of Iodine.*

| | | |
|---|---|---|
| "℞. | Iodine . . . . . . . . | ʒiv. |
| | Hydriodate of potash . . | ℥i. |
| | Distilled water . . . . . | ℥vi. |

"The solution should be kept in a bottle with a glass stopper, as it rapidly corrodes corks.

"This solution is very useful in cases where scrofulous surfaces require stronger excitement than usual, for example, to the eyelids and angles of the eyes in obstinate chronic ophthalmia, in coryza, or other forms of scrofulous disease in the nasal fossæ. It is most conveniently applied by means of pledgets of fine charpie. Even after a cure, I have frequently applied this solution to the deformed cicatrices characteristic of this disease, and it usually renders them smoother, less prominent and livid.

"The rubefacient solution may also serve two other important purposes, namely, the preparation of local baths and ioduretted poultices." (P. 172.)

The ioduretted baths have been found singularly serviceable by M. Lugol, and he warmly recommends them to be used in the treatment of many cases. He finds that wooden vessels are the best for containing the water impregnated with iodine; and the translator has given a very convenient table indicating the proportions of the several ingredients.

We cannot close this volume without again expressing our gratitude to Lugol for his unwearied exertions, by which he has wrought so much good; and if the use of iodine in scrofula did not originate with him, he at least has made the subject his own by the success with which he has crowned it: neither can we lay down our pen without offering our best thanks to Dr. O'Shaughnessy for giving these memoirs to the profession in their present condensed and improved form; indeed the author is under considerable obligation to him for the sound discretion he has used in abridging various prolix details, and in moderating exuberant passages, which, however excusable they are in the writer, and consonant with the genius of his tongue, seem somewhat strange to our more sober style, and little consonant with the rigidly didactic tenor of English medical details.

---

*On Polypi of the Heart as an Idiopathic Affection, and as a Cause of Death.* By William Harty, m.d., Physician to the King's Hospital, and to the Prisons of Dublin. (*Dublin Medical Transactions*, vol. i. part i.)

(Concluded from page 58.)

Case II. Master M., aged thirteen, had symptoms of measles, of a severe character, December 13th, 1818, followed by inflammation of the trachea, with painful and laborious respiration. Palpitation soon came on, and did not cease till death took place. Dyspnœa at times very distressing, from mucus in the trachea; started up suddenly from his sleep. On the evening of the day when the tracheal inflammation attacked the patient, the face was œdematous and livid. From repeated bleedings, and other appropriate remedies, the distressed respiration was relieved, but only for a time.

On the 19th, eruption of measles had disappeared, but there was œdema of the face and *slight emphysema* of both arms, and now the pulse began to impart the *peculiar sensation* described in the former case.

20th. Pulse 132, with the same rapid thrilling action as of a fluid rushing through a tube it did not fill. This sensation was imparted by every artery, however small, and felt "even through the thick muscles of the extremities." Face œdematous; arms and thorax emphysematous.

Until the evening of the 24th, when the patient expired, the principal symptoms that existed were hurried respiration; dyspnœa; distention of the alæ nasi; palpitations; pain and soreness at the region of the heart; occasional agitation and delirium.

The post-mortem examination was made in the presence of Dr. Crampton and Messrs. Meredyth and Harrison, and it is proper to observe, that, *before* the dissection, Dr. Harty "felt no hesitation in saying that an organized polypus would be found in the heart," from his recollection of the analogous case of Miss R. The morbid appearances are thus described.

"There was a considerable depression of the sternum, forming an arched hollow between the mammæ; a fact obvious to the eye during life. There was also unusual thickness of the sternum from the point of elevation upwards; there was slight adhesion of the left lung to the pleura in one or two points; also a slight degree of adhesion near the junction of the two lobes; the external coat of the pericardium was evidently inflamed, though not to any great degree or extent, and there was about an ounce of serous fluid in the pericardium. The liver was enlarged in its dimensions, though by no means to the same extent as in the former case; it was otherwise apparently healthy, while the heart itself appeared larger than was compatible with his years, and the general structure of his frame. The left ventricle and auricle of the heart contained a large and singular polypus, unconnected with any coagulum, and adhering firmly in some parts, and more loosely in others. In the auricle (properly so called,) it adhered firmly throughout, maintaining a perfect union therewith by a number of lateral projections, and thence descending into the ventricle by a long and narrow neck, it formed a flat and firm adhesion to the side of the ventricle, throwing out at the same time a band, whereby it was connected to the polypous concretion which loosely occupied the apex and body of the ventricle, and extended thence into the aorta. The body of the auricular polypus branched largely into the pulmonary veins, and in its thickest portion contained a distinct, dense, and compact clot of blood, enveloped therein." (P. 238.)

In these cases there were many symptoms which are common to various affections of the heart; but that which seemed to Dr. H. *peculiarly* to characterise the disease in each was that "singular thrilling, whizzing sensation, which every artery in the body, as well as the heart, imparted to the touch; and it is not a little remarkable that this sensation was first noticed in each on the *fifth* day before death." This symptom Dr. Harty considers the great diagnostic sign of the existence of polypus in the heart, or at least in the left ventricle. When it does occur, he believes that the polypus has entered the great vessels issuing from the heart, and that thus the action of the valves and the free flow of blood are obstructed. From this view of the subject, therefore, if correct, it would follow that polypus may exist in the heart without this diagnostic sign, *provided* it has not entered the valves.

"There are three other remarkable symptoms in which these cases agreed, but how far they are to be deemed diagnostic, I will not undertake to decide: there was in both, and at an early period, œdema of the face; there existed in both, even when the disease was most severe, the power of lying perfectly horizontal without any distress, and also of lying on the left side; when previously they could not do so; and there prevailed in both (so far at least as came under my observation,) from the beginning to the termination of the disease, the most perfect regularity of the pulse.* In both

* In the interesting case related by Dr. Watson, which we give at p. 513, the same fact was observed: "the pulse was at no time irregular."—Editor.

there was early, as also occasional delirium throughout the disease." (P. 242.)

Dr. Harty has notes of several cases, in which death occurred very suddenly, and in the subjects of which organized polypi were found in both ventricles; but as in these instances the diseases presented a very complicated aspect, he mentions them merely for the purpose of observing, that they all occurred about the same period either with that of Miss R. or Master M., thereby illustrating a conjecture of Laennec, "que peut-être même l'influence de la constitution regnante contribue-t-elle a leur formation autant que l'etat particulier du sujet. J'ai remarqué au moins que dans certain temps on en rencontre beaucoup plus frequemment de tres volumineuses." In confirmation of this conjecture, Dr. Harty refers to a singular detail given by Dr. Chisholm, in the fifth volume of the Annals of Medicine, of what he calls an "epidemic polypus;" and also to a paper by Dr. Huxham, in the Philosophical Transactions, No. 464, p. 123, giving an account of a number of sailors who died of polypi of the heart, after returning from a hot climate to England, in the severe winter of 1742. Another interesting case is also mentioned, which is recorded in Simmons' London Med. Journal for 1785: it is related by Mr. Cheston, of Gloucester, a well-known surgeon of that period. The subject of this case, Mr. Holder, was a surgeon-apothecary, in large and laborious practice: for four years before the fatal termination of his malady, in 1775, he had experienced occasional pain and distress in the thorax on any great exertion. A few months before death, the pain extended to the right side and up to the neck, where he was constantly sensible of a noise similar to that of a stream of water passing over obstructions, or forcing a passage through a narrow confined place. The gushing noise in the thorax became so strong and loud that he often expressed his surprise at its not being heard. Pulse was hard and contracted, and without intermission: it was singular that the motion of the heart was not to be felt, though repeatedly searched for. On dissection, the right auricle was found much enlarged, and very thin, "with strong marks of inflammation," and the right ventricle nearly transparent: on opening them, they were totally void of blood, and a "considerable quantity of air rushed out." At the upper part of the right ventricle, from between the columnæ carneæ, there arose a broad concretion, adhering firmly to the columnæ, about the thickness of half a crown, of a light yellow colour, very dense consistence, and occupying about two thirds of the diameter of the cavity; then it rose into the auricle, and thence nearly four inches into the vena cava superior, one into the inferior, and also into the pulmonary artery; the branches not so firm as the main body. Another small concretion, of darker colour and more tender consistence, arose from the columnæ carneæ of the left ventricle, passing into the aorta about one inch and a half. Mr. Cheston concludes the above case with some valuable observations on polypi of the heart, and mentions that in two instances he had met, where the peculiar symptom of "a noise in the ear like the dashing of water" had been described by the patients, he afterwards found a firm concretion in the right ventricle. Mr. C. adds, that there is every reason to believe that the concretion found in Mr. Holder's case was the source of the distressing sensations he felt; especially when we recollect "the noise so remarkable in aneurismal varix, as well as that described by patients under arterial dilatation, in which coagula are generally met with."

Dr. Harty states, in concluding his paper, that he is unwilling to deduce any positive conclusions from data so few in number; but he asks whether we *now* can agree with Burns, that "we have great reason to doubt whether a case of idiopathic polypus of the heart has ever occurred."

We are indebted to Dr. Watson, physician to the Middlesex Hospital, for the detail of the following very interesting case. The boy was a patient of Dr. Francis Hawkins, and Dr. Watson's attention was directed to him during the absence of his colleague from London. No doubt can be entertained that, in this instance, the fibrinous concretions which were found in the heart had existed for a long time before death; but whether this case can be adduced as one of "idiopathic polypus," we will not venture to determine.

"On the 2d of June, 1830, the body of Charles Roots, a boy twelve years old, who had died in the Middlesex Hospital, was examined.

"He had been a patient in the hospital one month, affected with general dropsy, palpitations, and pain in the situation of the heart. At the time of his admission, he was in a state of severe salivation. It was stated by his friends, that he had then been ill two months; that his legs first swelled, and the pain in the cardiac region succeeded; and that he had never had rheumatism. The action of the heart, as heard through the walls of the chest, was tumultuous, not very extensively audible, and without any *bruit*.

"For the first fortnight after his admission, he was very drowsy: subsequently he was prevented from sleeping much by severe pain about the heart, returning in paroxysms, and causing him to cry out. During the last four days of his life, the palpitation and pain had greatly subsided, and his chief distress arose from the distention of his legs and thighs. His legs were punctured, with some temporary relief, the fluid flowing from them copiously. The urine was never very deficient, and for the last two days he had passed it in abundance. For at least five days before his death, the external jugular vein, on the left side, appeared to the sight and touch as if choked up by a coagulum, and was acutely sensible to the slightest pressure. The pulse was at no time irregular, nor had there been any orthopnœa.

"The examination took place fourteen hours after death. It was prognosticated that polypus concretions would be found in the heart and jugular vein. There was a considerable quantity of serous fluid in the pleura, pericardium, and peritoneum, and in the cellular tissue of the body generally. The lungs were sound in structure, but gorged. The heart was much larger than natural; the left ventricle was much thickened, and slightly dilated; the right ventricle large and thin: the right auricle felt hard, and was lumpy or irregular externally: it contained a mass of white fibrinous concretion, which adhered to its inner membrane, and from which there burst, when it was pressed between the fingers, a quantity of matter resembling pus. This matter had been confined in the substance of the concretion, in a membranous cyst, of which the internal surface presented an irregular rugged appearance, very like that of a mucous membrane; its external surface could easily be separated by peeling, from that part of the clot with which it was in contact. The concretion adhered to the lining membrane of the auricle by numerous reddish fibres, and, when forcibly detached from this membrane, it left it studded with minute red points. There were several smaller cysts imbedded in the same coagulum, and also containing what seemed to be pus. Some of them were shelled out from the coagulum unbroken, and were afterwards laid open and emptied. These cysts were tolerably firm, and possessed considerable elasticity; standing open when empty, like an artery, but less firmly. In the right ventricle there was a large white fibrinous concretion, unattached to the interior of the ventricle, and exactly like those which are so often seen in the same situation: there were also a few separate concretions, varying in size from that of a peppercorn to that of a large garden pea, and each adhering to the lining membrane of the ventricle. In the left ventricle there was a small portion of coagulated blood, which, however, was not (like the former) white, but red. Among the reticular meshes formed by the columnæ carneæ of this cavity, there were several white bodies, of a round form and smooth surface, and of the size of those seen in the right ventricle: they also had evidently been formed of coagulated blood. Each contained a cyst full of pus, and each was adherent to the inner membrane of the ventricle, in the manner already described. There were likewise a few in the left auricle.

The left external jugular vein contained a large and long clot, which was easily pulled out from beneath the clavicle; together with a similar one from the internal jugular. The clot was partly red, and partly white, in no degree organized, or attached to the vessel. There was no apparent thickening or disease of these veins. The valves of the heart were all sound. The arch of the aorta seemed slightly dilated, and the descending aorta small when compared with the heart, though it was probably of the natural proportions when compared with the general bulk and stature of the body.

"The liver was not healthy; the kidneys were very large: their cortical part, instead of being of a dark colour, was of a light yellow, nutmeggy appearance, and granular, just like some of those described and represented by Dr. Richard Bright."

We give a translation of the following case from the Dict. des Sciences Med., t. v. p. 506, *Paroxysmal Disease of the Heart, arising probably from the Existence of Fibrinous Concretions.* L. W., æt. thirty-eight, was struck on the chest with a stick, in January 1804: extensive ecchymosis was produced, and severe pain and difficulty of breathing came on, which prevented the patient from working for three months. About five weeks after the infliction of the blow, the patient was suddenly attacked with violent palpitations of the heart, accompanied by sweating and difficulty of breathing. From this time similar palpitations returned occasionally, sometimes at intervals of three or four days, and at others a month intervened. These palpitations came on suddenly, and at the commencement of each attack were very violent. The most severe paroxysms had never lasted longer than one day, until the month of September, and it is remarkable that in the intervals the patient's health had been very good. At the beginning of September, the palpitations became almost constant; respiration impeded, and more difficult in a horizontal position; the pulsations of the heart were accelerated, and felt beyond the usual limits; pulse quick; urine scanty and red. In this state the patient continued for about three days; on the fourth, a sudden aggravation of all the symptoms occurred, and he died.

*Dissection.* Heart very large; right auricle and ventricle much dilated, and their parietes thickened and in a state of active aneurism; left auricle equally thickened, and hard and somewhat hypertrophied: it contained many fibrinous concretions, of a red colour, and attached so firmly to the internal surface of the heart, that they were separated with difficulty. It was evident that these concretions had existed for a long period. Left ventricle was not augmented in its capacity in proportion to the other cavities. Aorta rather large, rough and wrinkled internally.

By the older writers, polypus of the heart was considered a very common and a very fatal disease. This doctrine has met with few supporters among the medical authorities of modern times, and no doubt it was once carried too far: but we incline to the opinion that the more carefully the subject is investigated, the more reason shall we have to believe that polypi of the heart occasionally exist without any accompanying disease of the heart itself; that they may give rise to a severe train of symptoms during life, and ultimately cause the death of the patient. Whether there are any distinctive symptoms by which the existence of polypi of the heart can be determined, is a point that requires additional evidence.

*A Treatise on the Diseases of the Heart and Great Vessels, comprising a New View of the Physiology of the Heart's Action, according to which the Physical Signs are explained.* By J. Hope, M.D., Member of the Royal College of Physicians, &c.—8vo. pp. 612; seven diagrams. W. Kidd, London.

(Concluded from page 522 of the last volume.)

We will now take a glance at what may be called the more practical part of the work; that, namely, which treats of individual diseases; and we believe that in this, and in a few extracts which we shall present as *echantillons*, our readers will find proofs that the treatise to which we now call their attention is a production of no ordinary value and importance. The second part comprises inflammatory affections, namely, pericarditis, carditis, inflammation of the inner lining membrane of the heart and arteritis.

*Pericarditis.* The anatomical characters of acute pericarditis are, 1st, preternatural redness of the membrane; 2d, coagulable lymph adhering to its surface; 3d, fluid effused within its cavity. These several characters are treated of at some length, and the pathological effects of effusion and adhesion traced with considerable ingenuity.

"Supposing that the inflammatory process does not terminate by resolution, by the complete absorption of both lymph and serum, the most desirable termination which remains is adhesion; for, should this not take place, the lymph becomes a secreting surface, which effuses more and more lymph and serum, until, in a short time, the cavity is completely distended, and the action of the heart so embarrassed that a fatal termination speedily ensues. But, should adhesion of the opposite surfaces take place, by which further effusion is prevented, life may be prolonged for a considerable period, even for years; though, as will presently be explained, the adhesion, so far from being a perfect reparation, gives rise to another form of organic disease, which eventually proves destructive to the patient." (P. 88.)

The several varieties of adhesion that are described produce a considerable variety of symptoms and effects, according to their situation, and as they, on the one hand, restrain the effusion of fluid, and, on the other, embarrass the motions of the heart. Fluid effusions are easily reabsorbed; but when accompanied by an exudation of lymph on the membrane, they are perpetuated by continued secretion from the new surface, and gradually become milky, and more of the character of pus.

"*General signs.* There is no inflammatory affection of which the diagnosis has been considered more difficult than pericarditis. Laennec states that he has often, on dissection, discovered the disease in a severe form, when nothing had afforded a suspicion of its existence; and, on the other hand, that he has frequently witnessed all its signs, without finding a vestige of the malady. Dr. Latham mentions two cases of what appeared to be, and was treated as, marked inflammation of the brain; yet this organ was found perfectly sound, and the heart affected with intense pericarditis. Andral relates a similar case. It is proper to keep these difficulties prominently in view, in order that practitioners may be better prepared to contend with them: but it must be added, that such cases as those of Latham and Andral are extremely rare; and that, with the improvements in diagnosis introduced by modern research, the disease may, I feel assured from numerous post-mortem examinations, be nearly always detected." (P. 97.)

Then follows a very full and graphic description of the general signs; on the variable and apparently incongruous nature of which our author remarks, that, although they thus fail to be pathognomic, they are valuable indications in denoting the nature and progress of the changes of structure, and, in correspondence, the progress and exact state of the malady. The physical signs, when taken in conjunction with those general ones that do exist, are, however, always sufficient to detect the existence of the disease, if the attention is once drawn to them; which it would not fail to be, if, as Dr. Hope recommends, it were the general practice to place the hand on the precordial region, as well as on the pulse, in every severe inflammatory or febrile affection, as we daily feel the abdomen in cases of fever, although the patient make no complaint of it.

The physical signs of pericarditis are carefully described. Chronic pericarditis is often more obscure than the acute form, but the history generally affords the greatest light.

Dr. Hope, in accordance with Corvisart and preceding authors, and contrary to Laennec and Bertin, ranks adhesion of the pericardium among the bad, and ultimately fatal effects of pericarditis. Dr. H. mentions three physical signs by which adhesion of the pericardium can be recognised.

We pass over a very descriptive and interesting chapter on Acute and Chronic Inflammation of the Arteries and Internal Membrane of the Heart, as we could not do it justice in an abridgment, and our limits forbid many more extracts: the subject is one of much importance, as it includes that of the origin of valvular disease and various organic lesions of the arteries.

The chapter on Hypertrophy commences by remarks on the classification of the different kinds: Dr. H. names them as follows.

"1. Simple hypertrophy, in which the walls are thickened, the cavity retaining its natural dimensions.

"2. Hypertrophy with dilatation. This, the eccentric or aneurismal hypertrophy of Bertin, presents two varieties: viz.

"a. With the walls thickened and the cavity dilated.

"b. With the walls of natural thickness and the cavity dilated: i. e. hypertrophy by increased extent of the walls.

"3. Hypertrophy with contraction. In this the concentric hypertrophy of Bertin, the walls are thickened and the cavity is diminished." (P. 178.)

He adopts Laennec's description of the proportional size of the heart, and proceeds to describe minutely the mode in which hypertrophy affects it.

"When all the cavities are hypertrophous and at the same time dilated, the heart attains a volume, two, three, and occasionally even four times greater than natural; its form, instead of being oblong, is spherical; its apex is scarcely distinguishable, and, as the diaphragm does not retire sufficiently to yield space downwards for the enlarged organ, it assumes an unnaturally horizontal position, encroaching so far upon the left cavity of the chest as sometimes to force the lung upwards as high as the level of the fourth rib, or even higher. I lately examined a subject in which it had been forced much higher." (P. 182.)

The walls of the left ventricle are most liable to hypertrophy, and may be increased to double or treble their natural thickness. In the right, the columnæ carneæ become more enlarged. Hypertrophy of the auricles is rare, and almost always the species with dilatation. Dr. H. looks on hypertrophy of the heart as an instance of increased size, in consequence of increased action and augmented afflux of blood in the muscle, and attributes the greater liability of the strongest part, the left ventricle, to the same principle that hollow muscles resist over-distension in proportion to sheir strength.

"The left ventricle, for example, being charged with the immense burden of the greater circulation, is proportionably substantial and robust; the right, having the comparatively light task of propelling the blood through the minor or pulmonary system, is little more than one third as thick and powerful as the left: the auricles, again, having a still less laborious function to perform, have a still more limited muscular provision.

"Hence it is easily understood how a distending force sufficient to overcome the contractile and elastic power of the right ventricle, might merely operate as a stimulus to the superior muscularity of the left. While the former, therefore, incapable of reacting on its contents, would dilate; the latter, excited to extraordinary efforts, would become hypertrophous." (P. 189.)

The author, therefore, and for other reasons, discards Bertin's explanation, that the greatest tendency of the left ventricle proceeds from the more stimulating quality of the arterial blood. It appears, then, that every circumstance capable of increasing the action of the heart for a sufficient length of time, may be a cause of hypertrophy.

A section is devoted to explain the order of succession in which the several parts of the organ are rendered hypertrophous by an obstacle before them in the course of the circulation; and, as throwing light on the origin and progress of the disease, as well as on its effects on other parts of the circulation, the author's remarks are replete with interesting matter.

It is quite obvious that a disease which affects the function of so important an organ as the heart cannot exist without, in a notable manner, causing disorder in other parts of the system, the health of which so much depends on the regularity of the circulation. Laennec, although aware of this, yet carrying with him, in his remarks on hypertrophy, his uncertainty respecting the distinctive signs of the various diseases of the heart, did not recognise any as peculiar to this affection; which Dr. H. attributes to this pathologist and the preceding writers not having sufficiently analysed the cases; they studied them in the complications in which they usually occur. Bertin perceived this; but our author thinks that he goes too far in ascribing exclusively to the coexistent lesion the whole signs of disordered circulation, such as dyspnœa, engorgement of the capillaries, serous infiltration, &c., and in maintaining that the only effect of hypertrophy is to give increased energy and activity to the circulation.

"The truth I believe to be, that the very same energy of the circulation which gives rise to active hæmorrhages, apoplexy, &c. causes, as its next effect, engorgement of the arterial capillary system; the necessary consequence of which is, serous infiltration and more or less of all the other symptoms indicative of retardation of the blood. The process appears, in fact, to be analogous to that by which serous infiltration is produced in cases of erysipelas, scarlatina, acute rheumatism, &c. I would not be understood by this to mean that capillary congestion is identical with inflammation, but that, as the effects of the two are sometimes the same, we are compelled to admit a close analogy in the mode of their production." (P. 204.)

After several arguments in support of this opinion, the author concludes,

"The sum, then, of all that has been said, is, that pure hypertrophy gives rise to increased force and activity of the circulation, and that, when this force surmounts the natural tonic power of the capillaries, congestion, infiltration, and the other phenomena of an obstructed circulation, ensue." (P. 207.)

The effects of hypertrophy of the left ventricle in giving rise to apoplexy, and other diseases of the head, are very signal. Eight or nine cases of suddenly fatal apoplexy, and many of palsy, have, within a few years, fallen within our author's observation; and he is led to conclude that hypertrophy of the left ventricle forms a stronger predisposition to apoplexy than the apoplectic constitution itself. On this point our experience leads us to concur with our sagacious author. The history of individuals affected with hypertrophy of the left ventricle, moreover, frequently presents a striking narrative of violent headachs, brain fevers, and great states of nervous irritability from the same cause. Hypertrophy of the right ventricle frequently produces corresponding diseases in the lungs, as pulmonary apoplexy and active hemorrhage.

Of the general and physical signs of hypertrophy, Dr. H. says, "According to my experience, neither of these classes of signs, taken separately, is sufficient to indicate disease of the heart with perfect certainty: taken conjointly, they render the diagnosis so easy, that a material error can scarcely be committed." (P. 213.)

The general signs are described at great length. Of the physical signs, a strong, slowly heaving impulse is the most remarkable in simple hypertrophy; and when this is followed by a sinking back in a sudden manner, or forcible *back stroke*, as Dr. H. calls it, the disease may be known to be extensive. The sounds are diminished, unless in the common case of combination with dilatation, in which the signs become compounded with those of the latter disease. These peculiarities, and the causes on which they depend, are then stated, and are in uniformity with the pathology developed in the preceding sections.

*Treatment.* Hypertrophy is, more than any other organic disease of the heart, susceptible of cure. The plan of treatment recommended by our author is of the moderately antiphlogistic kind: he objects to the extreme method of Valsalva, on account of the repeated and excessive bloodlettings giving rise to reaction and palpitation, which defeat their end and exhaust the patient. From four to eight ounces of blood, taken every two, three, four, or six weeks, according to the strength, are sufficient to keep down palpitation and strong impulse of the heart, without exciting reaction. The diet should be light and nourishing. Dr. H. has seen the most decided advantage from the use of diuretic drinks, such as cream of tartar and broom tea, which drain off the serous portion of the blood. Decided dropsy must be combated by more active diuretics. Sedatives are also often necessary to tranquillise the action of the heart; and for this purpose tincture of digitalis, hydrocyanic acid, hyoscyamus, conium, and acetate of morphia are, in different cases, eligible. The treatment recommended must be steadily pursued one, two, three, or more years; and thus employed, Dr. Hope has known it effect cures in many instances, some even advanced to the second degree. In the first degree, particularly before the age of puberty, cures may sometimes be effected with fewer bleedings, as once in from six weeks to three months.

An important chapter on Dilatation next follows.

Softening and Induration, Accidental Productions, Degenerations, and Atrophy, next occupy successive chapters, which we must pass over to notice the important and interesting one on Diseases of the Valves and Orifices. This occupies nearly eighty pages, and contains a great deal that is original and of momentous practical relations. After a full description of the anatomical characters and varieties of valvular disease, indurations and vegetations, the author proceeds to notice their pathological effects, which he represents to depend, in a very important degree, on circumstances.

"It is of immense practical importance to keep in view the facts stated, namely, that valvular contraction does not produce formidable symptoms until it has given rise to hypertrophy or dilatation; and that it invariably leads to these affections unless the circulation be kept tranquil. We thus know that the most efficacious treatment of valvular disease consists in employing such prophylactic measures as are calculated to prevent the supervention of hypertrophy or dilatation, and employing them with the same uncompromising strictness before those affections have appeared, as if they actually existed." (P. 326.)

The importance of this distinction needs scarcely to be dwelt upon: on this point Dr. Hope further remarks,

"From what has been said here and in the chapter on dilatation, the reader will judge how totally some authors have been wrong in referring the obstruction of the circulation to the valvular contraction exclusively, without allowing that the enlargement of the heart contributes in any degree to the effect. Such a doctrine is not only erroneous, but dangerous, as it leads to pernicious practice; for, imagining the valvular contraction to be the only formidable part of the complaint, to it alone those authors direct their attention; and, acting on the inaccurate presumption that it is in all cases caused by, and accompanied with, inflammation, they attack it with bloodletting, general and local, abstinence, digitalis, &c.; means which cannot remove valvular contraction when once formed, (as must always be the case before the symptoms can exist,) and which are, therefore, a useless expenditure of the patient's strength: it is true, indeed, that measures calculated to diminish the force of the circulation are useful in obviating the supervention of hypertrophy or dilatation, the paramount source of danger in these cases; but measures employed for this purpose, and which must be continued for an indefinite length of time, cannot be practised with the same activity as for the purpose of curing an inflammation. I would not be understood by this to mean, that valvular disease is never accompanied by inflammation, and that, when so accompanied, it should not be treated by antiphlogistic measures; but I mean that they should not be employed unless there is reasonable evidence of inflammation." (P. 327.)

The general signs of diseases of the valves are, 1, those of dilatation, but in aggravated form; as cough, copious watery expectoration, dyspnœa and orthopnœa, frightful dreams and starting from sleep, pulmonary apoplexy and passive hæmoptysis, turgescence of the jugulars, lividity of the face, general dropsy, engorgement of the viscera, and congestion of the brain, with symptoms almost amounting to apoplexy; 2, the signs peculiar to diseases of the valves, as various irregularities of the heart's action, palpitation, intermittent pulse, and pain in the region of the heart. This last symptom, although it may occur in palpitation with or without organic disease, is especially remarkable where either the valves, the coronary arteries, or the commencement of the aorta, have become indurated and unyielding. It is various in its intensity, and often shoots or lancinates to the scrobiculus cordis, and down the elbow. Dr. H. considers this pain, or angina, to be caused by the inelasticity of the indurated parts, which will not yield to the increased motion of palpitation. A pulse which is weak, intermittent, and unequal, whilst the action of the heart is violent and impulsive, affords one of the strongest presumptions of the presence of valvular disease.

Our author observes, that the accession of auscultation to the other means of diagnosis has rendered it possible to distinguish valvular disease, with almost complete certainty. This is an important assertion, and one which many, well versed in auscultation, have been unable to make. Laennec himself admitted the uncertainty of his favorite class of signs in this instance; but Dr. H. attributes this uncertainty solely to the errors in the views of the inventor of auscultation: he conceives that a contracted orifice or rigid valve, by throwing the blood into more than ordinary commotion, always occasions a sound or murmur; this sound varies according to the nature of the contraction, being soft and like the blowing of a bellows when the orifice is smooth, and harsh or grating, like sawing, rasping, or filing, when the morbid deposit is osseous and irregular. Murmurs are more hollow when deep-seated, as in the auriculo-ventricular orifices; and whizzing when superficial, as in the arterial commencements. The purring tremor (*fremissement cataire*,) seems to be of the same nature with the murmurs, but to depend on an increased force or velocity of the circulation, whether temporary or arising from hypertrophy; it may occur, therefore, without valvular disease. For the characteristic signs of valvular disease we must refer to the work.

Asthma is one of the diseases to which recent pathological researches have given a novel aspect, and which has by them been proved to be so varied in its nature, that the propriety of uniting the several affections which give rise to it under one term may well be questioned: it is, in fact, properly no more than a symptom, *dyspnœa coming on in paroxysms*; and this is the only general definition that can be given to it: all other characters are contingent, and depend on the pathological cause, and not on the asthma itself. Almost every disorder which interferes with the action of the respiratory organs may be the cause of an asthmatic paroxysm, but there are some pathological states of the thoracic viscera which are so frequently connected with it, as to have obtained themselves the name of asthma: such are the pituitous and dry catarrhs of Laennec, chronic bronchitis, pulmonary emphysema, and organic diseases of the heart. It is scarcely necessary to observe, that the adoption of such a name, if it be founded on the common symptom only, and in ignorance of the real lesion, may tend to blind and to mislead as to the primary cause; but this being known and acknowledged, there will be less objection to retain the term to designate the symptom: but there is a form of asthma which may more rigidly admit of the name, that depending solely on a spasmodic constriction of the bronchi; the existence of which we consider sufficiently proved by the observations and reasonings of Laennec. From the same researches, it has become a matter of high probability that secondary, or symptomatic asthma, is often the cause of this character; the spasm being excited in organs labouring under congestion, or other derangement. Violent exercise, or any thing capable of accelerating the circulation and respiration beyond a certain point, may produce this spasm, for a short time, in any healthy individual: when he is panting, for instance, after hard running, the sound of respiration in the chest is so weak as to leave no doubt that a temporary obstruction prevents the perfect ingress of air into the aircells.

The frequency of asthmatic symptoms in diseases of the heart has been often alluded to by recent writers on these diseases; but, as Dr. Hope observes, those who have treated of asthma, even of late years, have wholly omitted the consideration of these diseases as causes. To the magnitude of this error he is anxious to call the attention of the profession, and, speaking of asthma from this cause, observes,

"This variety comprises by far the greatest number of the most severe and fatal cases of the disease. Some are of opinion that in other varieties the patient experiences an equal degree of suffering during the continuance of the paroxysm. I cannot say that this is consistent with my own observation. Though the same words may suit for the delineation of an attack of each variety, my feeling and conviction is, that I have never seen the patient suffer such intense and suffocative agony as in the variety from organic disease of the heart." (P. 354.)

Dr. H. states that he has been often able to foretell the attack of a paroxysm by a palpitation or irregular action of the heart, perceived by auscultation; and he seems to consider this inordinate action of the heart as the primary derangement, in its turn exciting the bronchial spasm, which constitutes the proximate cause of the asthmatic paroxysm. Thus discarding the notion that a spasmodic or convulsive contraction of the external muscles of respiration causes asthma, he views this action as an instinctive and salutary effort to prevent suffocation; and in this view we entirely concur. The description which is given of asthma is powerful and forcibly drawn; we think too much so for the greater number of examples that we meet with in practice; and, if such a formidable array of symptoms, and such a hopeless termination, be among the necessary characteristics of cardial asthma, we should be inclined to dispute our author's assertion as to the proportion which this kind bears over others. How many cases do we meet with, even of the severest form during the paroxysm, in individuals that continue in other respects to enjoy robust health, and who, in spite of their occasional attacks, live on to an advanced age! Without disputing that the disease of the heart is sometimes primary, we confess that we incline to the opinion that it is, in many cases, a consequence of the obstructed and disordered circulation which attends the paroxysms of the spasmodic or bronchial asthma, whether simple or conjoined with other disease of the respiratory apparatus. Whether this opinion be correct or not, will not in any degree affect the importance of attending carefully to the state of the primary organ of the circulation; for, if this is diseased, no matter whether in the first or second place, this disease becomes the most pressing object of our care; for, besides that it is in itself dangerous and destructive, its combination aggravates the asthma, and gives it a new and much worse character.

"Asthma from disease of the heart often imitates the characters of the other varieties; and this perhaps for a very simple reason, that the lungs are in much the same state as in those varieties: thus, it is humid when there is permanent engorgement of the lungs, causing copious sero-mucous effusion into the air-vessels, as from contraction of the mitral valve; it is dry, when the engorgement is only temporary, as in cases of pure hypertrophy; it is continued when there is a permanent obstruction to the circulation; and any of the varieties may be convulsive when the heart has sufficient power to palpitate violently. The worst cases of convulsive asthma from disease of the heart, are those of hypertrophy with dilatation and a valvular or aortic obstruction." (P. 357.)

Bearing in mind the fact stated above, that valvular disease becomes formidable only when combined with hypertrophy or dilatation, the most important object in the treatment is to counteract the natural tendency which exists to the supervention of these.

"The remedies calculated to answer these indications are, in general terms, such as diminish the force and activity of the circulation; namely, occasional venesection to a moderate extent, an unstimulating and rather spare, though sufficiently nutritious diet, a tranquil life with respect both to the body and the mind, and a good state of the digestive organs and alimentary canal." (P. 366.)

"In general, if the valvular obstruction is not very considerable, and there is no hypertrophy or dilatation, and no tendency to plethora, an abstemious, light diet, and a scrupulously tranquil life, with an open state of the bowels, constitute all the prophylatic treatment that is necessary; and it is satisfactory to know that, by these means, danger may in many instances be completely averted. I have several times known patients with a moderate, even with a rather considerable valvular obstruction, attain the age of sixty, seventy, and even eighty, though the symptoms, judging from their account, had commenced early in life.

"On the other hand, if precautionary measures be neglected, and hypertrophy or dilatation superinduced, there is no organic disease of the heart, except adhesion of the pericardium, which tends more rapidly to its fatal termination. Hence the great importance of detecting and attending to disease of the valves in its earliest stage." (P. 367.)

In more advanced cases, however, unfortunately, little can be expected beyond temporary amelioration, and a delay of that fatal event to which the disease then inevitably tends. The particulars of the treatment, in conformity with these principles, are then entered into at full length; but for these we must refer our readers to the work itself.

We should be disposed to lay before our medical brethren some notice of several of the succeeding chapters, particularly those on Aneurism of the Aorta, Malformation of the Heart, Angina, and Polypus, which contain a great deal of original information of high interest and practical importance, as well as the instructive and illustrative selection of cases that terminates the volume; but our limits restrain us.

We here close our review, with the conviction that we have laid before our readers enough to enable them to form some judgment of the merits of this valuable work. Rising as we do from the perusal pleased and instructed, we feel, however, in duty bound with thanks to express our own opinion, that Dr. Hope has given us a far more complete treatise on Diseases of the Heart than any preceding writer; and we would place it beside Dr. Forbes' translation of Laennec, as a necessary appendage or supplement, to complete the parts which the illustrious Professor and his sagacious commentator had left imperfect, and forming, with that work, a standard and comprehensive history of the nature and treatment of diseases of the chest.

